# Effect of ultrapure lipopolysaccharides derived from diverse bacterial species on the modulation of platelet activation

**DOI:** 10.1038/s41598-019-54617-w

**Published:** 2019-12-03

**Authors:** Thomas M. Vallance, Divyashree Ravishankar, Dina A. I. Albadawi, Harry Layfield, Jonathan Sheard, Rajendran Vaiyapuri, Philip Dash, Ketan Patel, Darius Widera, Sakthivel Vaiyapuri

**Affiliations:** 1 School of Pharmacy, University of Reading, Reading, RG6 6UB United Kingdom; 2 Stem Cell Biology and Regenerative Medicine Group, School of Pharmacy, University of Reading, Reading, RG6 6UB United Kingdom; 3 Sheard BioTech Ltd, 20-22, Wenlock Road, London, N1 7GU United Kingdom; 4 School of Pharmacy, University of Reading Malaysia, Johor, Malaysia; 5 School of Biological Sciences, University of Reading, Reading, RG6 6UB United Kingdom

**Keywords:** Innate immune cells, Cardiovascular biology

## Abstract

Platelets are small circulating blood cells that play essential roles in the maintenance of haemostasis via blood clotting. However, they also play critical roles in the regulation of innate immune responses. Inflammatory receptors, specifically Toll-like receptor (TLR)-4, have been reported to modify platelet reactivity. A plethora of studies have reported controversial functions of TLR4 in the modulation of platelet function using various chemotypes and preparations of its ligand, lipopolysaccharide (LPS). The method of preparation of LPS may explain these discrepancies however this is not fully understood. Hence, to determine the impact of LPS on platelet activation, we used ultrapure preparations of LPS from *Escherichia coli* (LPS_EC_), *Salmonella minnesota* (LPS_SM_), and *Rhodobacter sphaeroides* (LPS_RS_) and examined their actions under diverse experimental conditions in human platelets. LPS_EC_ did not affect platelet activation markers such as inside-out signalling to integrin α_IIb_β_3_ or P-selectin exposure upon agonist-induced activation in platelet-rich plasma or whole blood whereas LPS_SM_ and LPS_RS_ inhibited platelet activation under specific conditions at supraphysiological concentrations. Overall, our data demonstrate that platelet activation is not largely influenced by any of the ultrapure LPS chemotypes used in this study on their own except under certain conditions.

## Introduction

Platelets (small, circulating blood cells) are responsible for blood coagulation upon vascular injury although their unwarranted activation leads to thrombosis. Platelets also play critical roles in the regulation of innate immune responses through diverse molecular mechanisms^1–4^. Toll-like receptors (TLRs) are a group of immune receptors that recognise pathogen- (PAMP) and damage-associated molecular patterns (DAMP)^5^. In humans, TLR4 is expressed in various immune cells and it plays critical roles in the regulation of inflammatory responses. The high affinity ligand for TLR4 is lipopolysaccharide (LPS), a molecule that is found in the outer membrane of Gram-negative bacteria^1,2^. TLR4 may activate two different signalling pathways (the MyD88-dependent or -independent pathway) depending on the ligand involved, as demonstrated in a glioblastoma cell line^6^.

It has been widely reported that TLR4 is functional in platelets^3,7^. Moreover, various signalling molecules involved in the MyD88-dependent and -independent signalling pathways downstream of TLR4 have been reported to be present in platelets^8,9^ which also emphasise the notion that TLR4 is functional in platelets. The presence of signalling molecules involved in both pathways suggests the potential binding of TLR4 with different LPS chemotypes to trigger either MyD88-dependent or -independent signalling^10^. Binding of *Escherichia coli* LPS (LPS_EC_) to TLR4 has been reported to increase the level of fibrinogen binding on the surface of platelets under arterial flow conditions^7^. Furthermore, circulating platelets have been reported to respond differently to diverse LPS chemotypes^11^. Moreover, it has been demonstrated that “rough (as referenced in the previous report – ‘without the O antigen’)” LPS [obtained from *Salmonella minnesota* (LPS_SM_)] is capable of enhancing platelet aggregation in PRP whereas “smooth (as referenced in the previous report - ‘with the O antigen’)” LPS (obtained from LPS_EC_) has no effect^12^. This is also reflected in “rough” LPS significantly inducing the release of platelet-derived microparticles on its own and with agonist whilst “smooth” LPS had no significant effect^12^. Diverse platelet responses such as platelet-neutrophil interactions^3^, fibrinogen binding^13^, and sCD40L secretion^14^ have also been observed following stimulation by LPS_EC_ although there is still no overall consensus on the LPS-induced effects in platelets^2^. For example, Claushuis *et al*.^15^ suggested that LPS_EC_ is only capable of influencing mitochondrial respiration in platelets although they have reported a significant increase in P-selectin exposure on platelets obtained from septic patients^15^. Furthermore, Koessler *et al*.^16^ recently suggested that the preparation of platelets is a factor in the response to LPS as it could only potentiate platelet responses in washed platelets and not in platelet-rich plasma (PRP)^16^. The reasons for the discrepancies of the results reported in the previous studies^7,13,15,17–19^ are unclear although the inadequate purification of LPS may result in the presence of bacterial contaminants, such as cell wall components that act as TLR2 ligands, and might be responsible for the controversial results^20,21^.

In order to determine the impact of LPS chemotypes obtained from various bacterial species on the modulation of platelet activation under different experimental settings, we used ultrapure LPS_EC_, LPS_SM_, and *Rhodobacter sphaeroides* (LPS_RS_, which is a TLR4 antagonist^13,22,23^) and analysed their effects in platelets. Here, we demonstrate the inability of ultrapure LPS chemotypes from various bacterial species to directly modulate platelet reactivity under diverse settings at physiological concentrations in contrast to conventionally prepared LPS.

## Results

### LPS_EC_ and LPS_SM_ induce NF-κB activity in a reporter cell line

**Figure 1:**
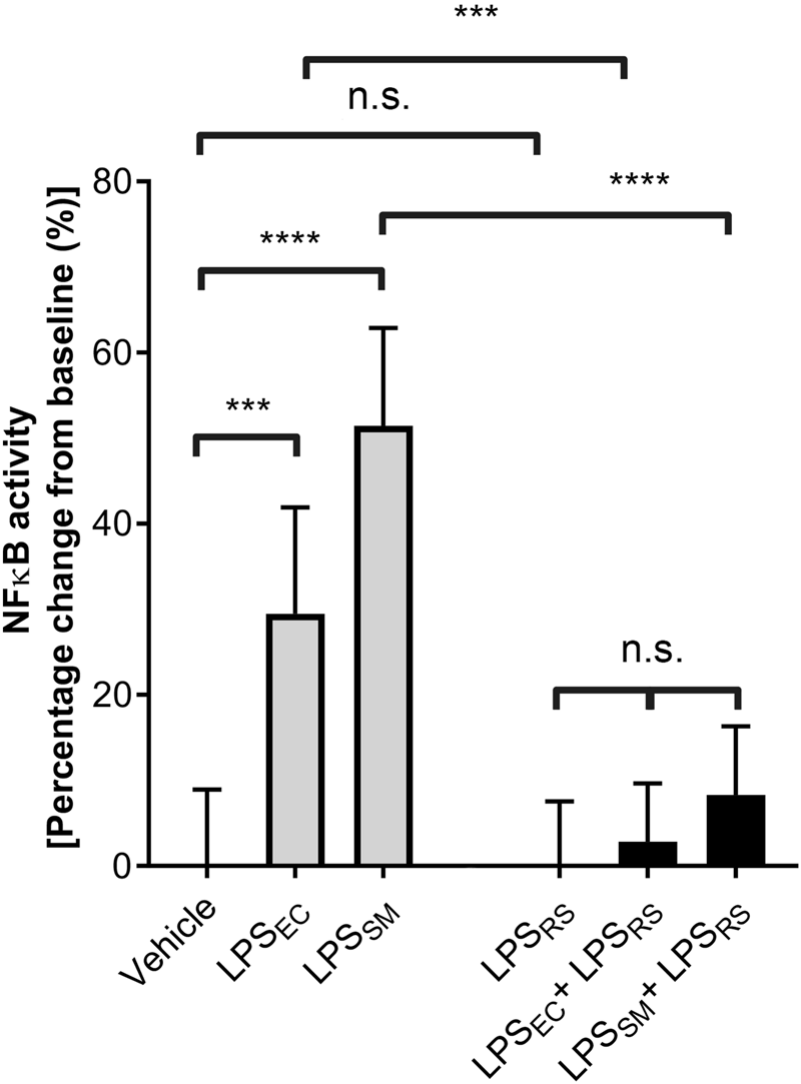
Effect of LPS chemotypes on the U251-NF-κB-GFP-Luc reporter cell line. After 3.5 hours of starvation, the cells were incubated with or without 10 μg/mL LPS_RS_ for 30 minutes. Then, U251-NF-κB-GFP-Luc cells were treated with 1 μg/mL LPS_EC_ or 1 μg/mL LPS_SM_ for 24 hours before lysis and measurement of luciferase activity by spectrophotometry. The results were normalised to the mean of vehicle control treated cells or the mean of the LPS_RS_ control. The data represent percentage change from control ± S.D. (n = 6). The *p* values shown are as calculated by a one-way ANOVA with Bonferroni’s post-hoc test (***p < 0.001, and ****p < 0.0001).

In order to confirm whether the ultrapure LPS chemotypes used in this study are functionally active and selective to TLR4, they were individually tested in U251-NF-κB-GFP-Luc cells, a reporter cell line for NF-κB signalling selectively via TLR4 (as they do not express TLR2)^24^. The results (Fig. 1) demonstrate that both LPS_EC_ and LPS_SM_ are able to significantly increase NF-κB activity whereas LPS_RS_ did not affect this activity (as expected for a TLR4 antagonist) in comparison to the controls. Notably, no significant increase in NF-κB activity induced by LPS_EC_ or LPS_SM_ was seen in the presence of LPS_RS_. These data suggest that the LPS chemotypes used in this study are capable of ligating to TLR4 and induce its downstream signalling, and that LPS_RS_ acts as an antagonist for TLR4.

### TLR4 is prominently detected in activated platelets

**Figure 2:**
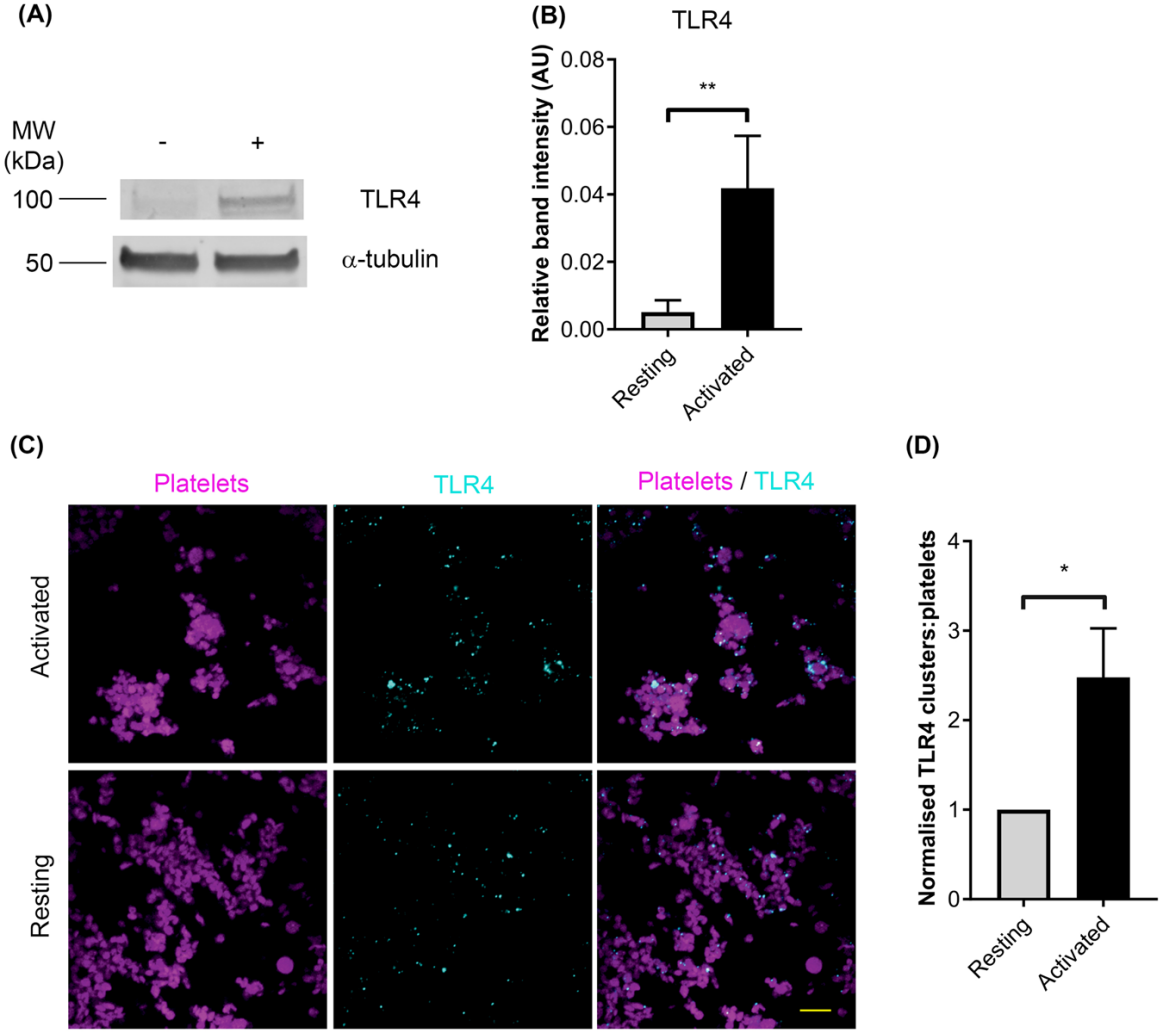
Expression of TLR4 in platelets. (**A**) Resting (−) and CRP-XL (0.5 μg/mL) activated (+) platelets were examined for the presence of TLR4 via immunoblotting. The level of α-tubulin was detected as a loading control. The blot shown is representative of five separate experiments using platelets obtained from five separate donors. (**B**) Quantification of the presence of TLR4 in platelet lysates compared to the expression of α-tubulin using immunoblots. Data represent mean ± S.D. and the *p* value was calculated using Student’s t-test (n = 5, **p < 0.01). (**C**) The level of TLR4 expression on resting and 0.5 μg/mL CRP-XL activated platelets was analysed by confocal microscopy using a 100x objective. The platelets are shown in magenta and the TLR4 is displayed in cyan. The images shown are representative of data obtained from three separate individuals. The scale bar represents 10 μm. (**D**) Quantification of the level of TLR4 in confocal microscopy images obtained from different donors. Three different regions of three images for each donor were analysed for the presence of TLR4 clusters and presented as mean ± S.D. *P* value (*p < 0.05) was calculated using Student’s t-test.

The presence of TLR4 on the platelet surface has been described previously and its level has been reported to increase following platelet activation^7,11,15,25,26^. To corroborate the presence of TLR4 in platelets, immunoblotting was performed using human isolated platelets that were stimulated with 0.5 μg/mL CRP-XL [a potent agonist acting via platelet glycoprotein VI (GPVI)]^27^. The presence of TLR4 was predominantly detectable in activated platelets compared to the resting cells (Fig. 2A,B). The selectivity of TLR4 antibody used was confirmed using U251-NF-κB-GFP-Luc cell lysates as a positive control and HEK293 lysates as a negative control (Fig. S1A). The increased level of TLR4 was also detected in platelets that were stimulated with 0.5 μg/mL collagen (Fig. S1B). Furthermore, the presence of TLR4 was confirmed through immunostaining of platelets (Fig. 2C) where a significant increase on the surface of platelets was observed upon activation with 0.5 μg/mL CRP-XL (Fig. 2D).

### LPS_EC_ does not affect platelet activation

**Figure 3:**
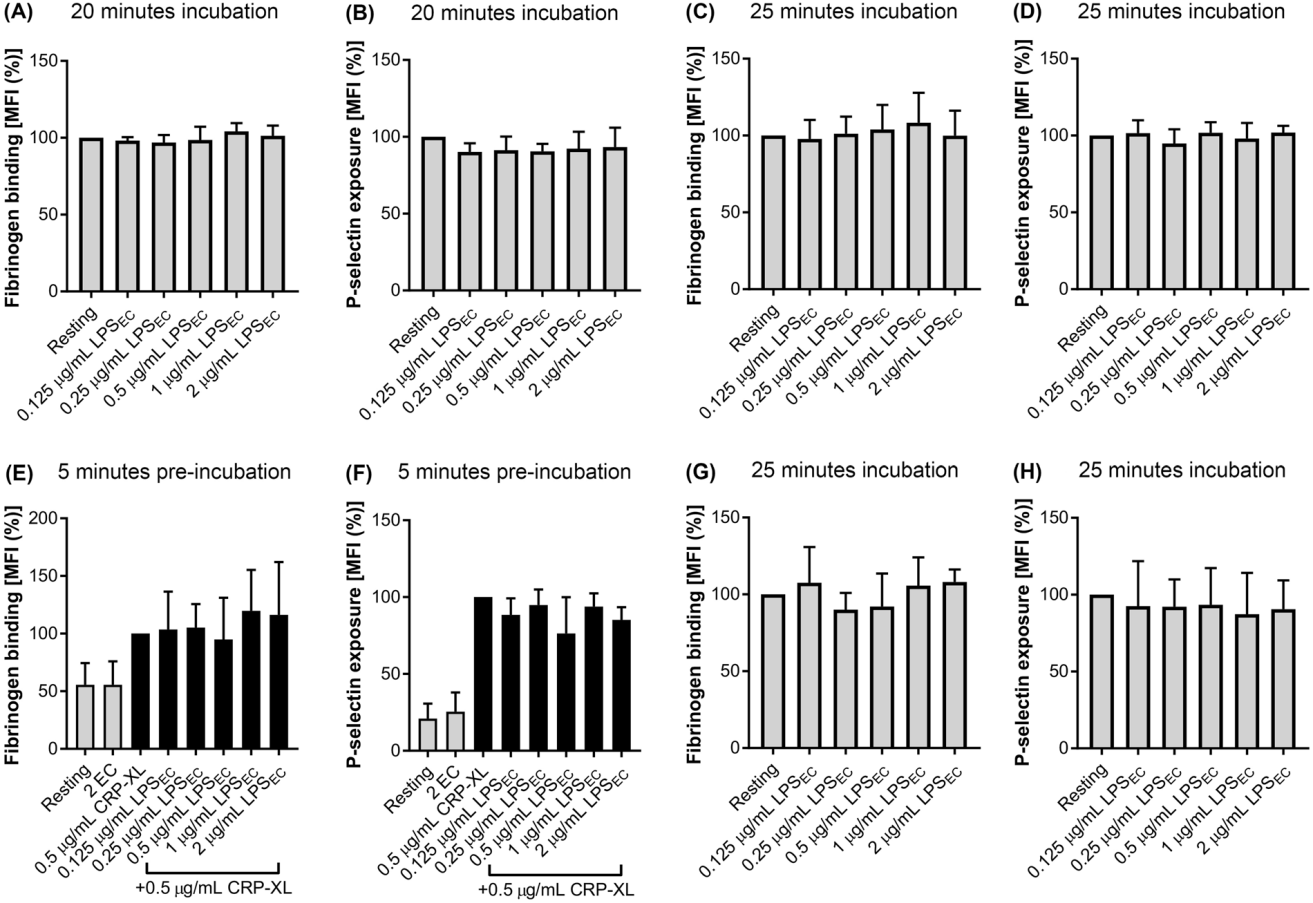
Effect of LPS_EC_ on platelet activation. The level of fibrinogen binding (as a marker for inside-out signalling to integrin α_IIb_β_3_) (**A**) and the level of P-selectin exposure (as a marker for α-granule secretion) (**B**) was measured in PRP upon incubation with LPS_EC_ for 20 minutes at room temperature (n = 4). In order to determine the impact of temperature on LPS-induced effects in platelets, the level of fibrinogen binding (**C**) and P-selectin exposure (**D**) in PRP was measured by incubating PRP with LPS_EC_ for 25 minutes at 37 °C (n = 5). To determine if the LPS chemotypes possess priming effects in platelets, the level of fibrinogen binding (**E**) and P-selectin exposure (**F**) was measured in PRP upon preincubation with LPS_EC_ for 5 minutes followed by stimulation with a vehicle control or 0.5 μg/mL CRP-XL for 20 minutes at 37 °C (n = 3). Similarly, the level of fibrinogen binding (**G**) and P-selectin exposure (**H**) in human whole blood was measured upon incubation with LPS_EC_ for 25 minutes at 37 °C (n = 3). The data were normalised to either their resting control (100%: **A**–**D** and **G**,**H**) or their 0.5 μg/mL CRP-XL control (100%; **E**,**F**) and analysed using one-way ANOVA and Dunnett’s post-hoc test. Data represent mean ± S.D. 2 EC - 2 μg/mL LPS_EC_.

To investigate the effect of different ultrapure LPS chemotypes on platelet activation, flow cytometry-based assays were performed to measure the levels of fibrinogen binding (a marker for inside-out signalling to α_IIb_β_3_^28^) and P-selectin exposure (a marker for α-granule secretion^1,29^). As TLR4 signalling in platelets was generally considered as pro-inflammatory, we hypothesised that LPS_EC_ may directly activate platelets. Therefore, LPS_EC_ was initially tested in platelets in the absence of an agonist^7,13,18^. The effect of diverse concentrations of (0.125 μg/mL–2 μg/mL) ultrapure LPS_EC_ on platelet activation was determined using human PRP or whole blood under different conditions such as various temperature and incubation times. The conditions tested were: incubation of PRP with LPS_EC_ at room temperature for 20 (Fig. 3A,B) or 25 minutes (Fig. S2A,B), and incubation of PRP with LPS_EC_ at 37 °C for 25 (Fig. 3C,D) or 50 minutes (Fig. S2C,D). These results demonstrate that LPS_EC_ does not induce platelet activation in PRP as measured by the levels of fibrinogen binding and P-selectin exposure at these conditions. To determine whether LPS_EC_ has priming roles on platelets as reported previously^30^, its effect on CRP-XL-activated platelets (Fig. 3E,F) was analysed through preincubating it for 5 minutes with PRP prior to stimulation with 0.5 μg/mL CRP-XL for 20 minutes at room temperature. Again, the LPS_EC_ failed to increase the level of CRP-XL-induced platelet activation. Furthermore, whole human blood was used to investigate the effect of LPS_EC_ on platelet activation in the presence of other blood cells but no significant change was observed (Fig. 3G,H). Together, these data demonstrate that ultrapure LPS_EC_ was unable to significantly increase either the level of fibrinogen binding or P-selectin exposure on human platelets under any of the conditions tested in this study. However, the non-ultrapure version of LPS_EC_ was able to significantly increase fibrinogen binding in platelets under similar conditions without the presence of a platelet agonist (Fig. S2E). In contrast, PRP from the same donors did not respond to ultrapure LPS_EC_ (Fig. S2E).

### LPS_SM_ modulates platelet activation at specific conditions

A previous report using U251-NFκB-A1 cells has suggested that ultrapure LPS_SM_ possesses the capacity to bias signalling mediated by TLR4 towards the MyD88-independent pathway^6^. In order to determine the effect of LPS_SM_ on platelets, various concentrations of this LPS were tested on the modulation of platelet activation upon stimulation with CRP-XL. Similar to above experiments with LPS_EC_, the levels of fibrinogen binding and P-selectin exposure were measured in platelets upon treatment with LPS_SM_ in the presence and absence of CRP-XL.

**Figure 4:**
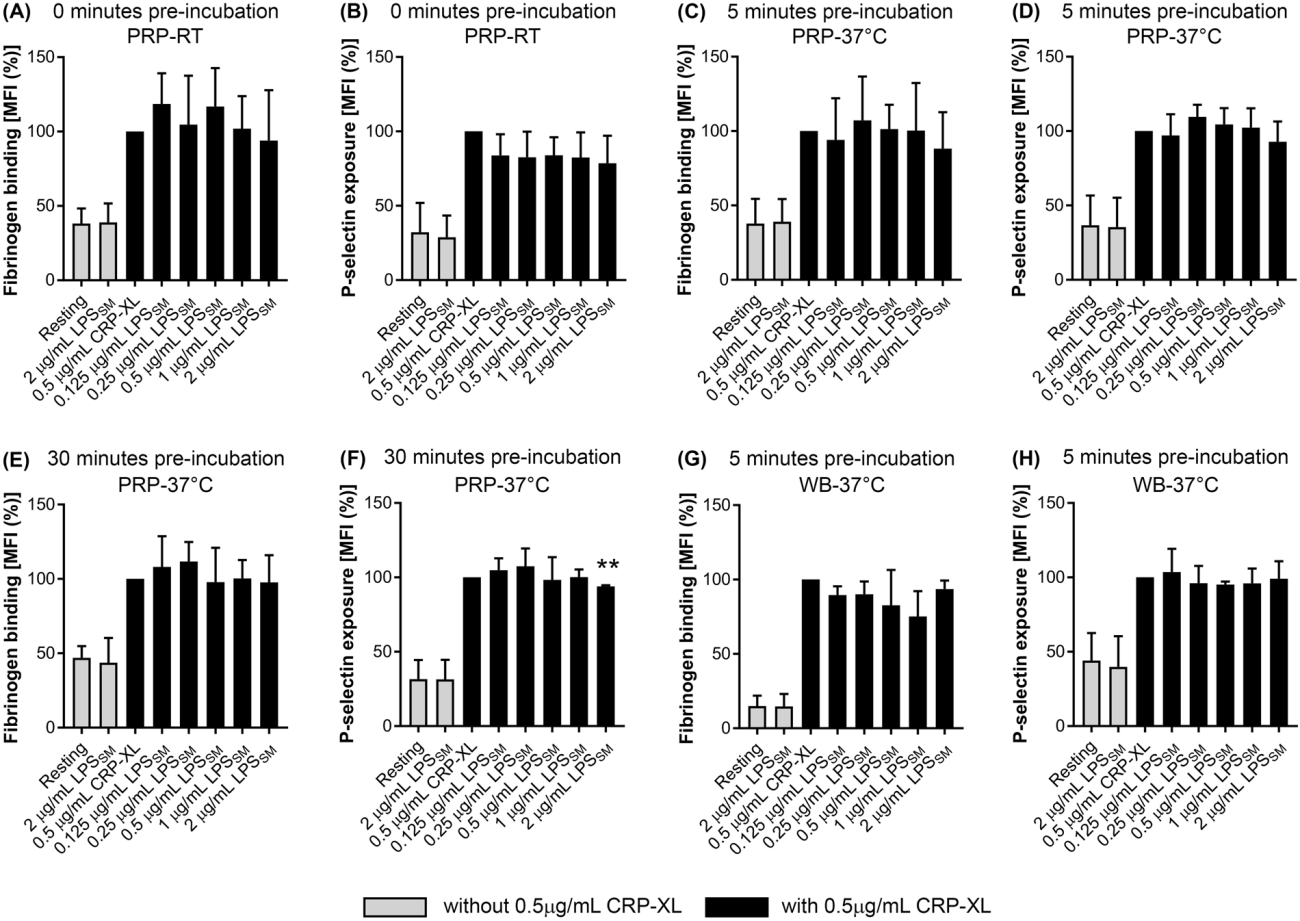
Effect of LPS_SM_ on platelet activation. To determine the impact of LPS_SM_ on the modulation of platelet activation, the level of fibrinogen binding (**A**) and P-selectin exposure (**B**) was analysed in PRP in the presence of different concentrations of LPS_SM_ and 0.5 μg/mL CRP-XL after simultaneous incubation for 20 minutes at room temperature (n = 5). To determine the impact of temperature on LPS-mediated effects in platelets, the level of fibrinogen binding (**C**) and P-selectin exposure (**D**) in PRP was measured upon preincubation with LPS_SM_ for 5 minutes followed by stimulation with 0.5 μg/mL CRP-XL for 20 minutes at 37 °C (n = 6). Similarly, the preincubation of PRP with LPS_SM_ was increased to 30 minutes prior to stimulation with 0.5 μg/mL CRP-XL for 20 minutes at 37 °C, and the level of fibrinogen binding (**E**) and P-selectin exposure (**F**) was analysed (n = 4). Furthermore, the level of fibrinogen binding (**G**) and P-selectin exposure (**H**) was analysed in human whole blood upon preincubation with LPS_SM_ for 5 minutes followed by stimulation with 0.5 μg/mL CRP-XL for 20 minutes at 37 °C (n = 3). The data were normalised to their 0.5 μg/mL CRP-XL control, and the *p* value (**p < 0.01) shown was calculated using one-way ANOVA and Dunnett’s post-hoc test. Data represent mean ± S.D. The samples treated in the absence of 0.5 μg/mL CRP-XL are represented with empty bars.

To determine the effect of LPS_SM_ on platelets in the absence of an agonist, PRP that was incubated with 2 μg/mL LPS_SM_ was analysed along with CRP-XL-activated platelets as a control in different experimental conditions. The results (Fig. 4A–H) demonstrate that LPS_SM_ does not instigate platelet activation in isolation.

To investigate whether LPS_SM_ has any modulatory effects on the agonist-induced platelet activation, platelets were treated with LPS_SM_ and 0.5 μg/mL CRP-XL simultaneously at room temperature for 20 minutes. These results (Fig. 4A,B) suggest that LPS_SM_ does not affect CRP-XL-induced platelet activation when they were treated simultaneously at room temperature. Subsequently, the platelets were stimulated with a low (0.25 μg/mL) concentration of CRP-XL (Fig. S3A,B) and LPS_SM_. Again, LPS_SM_ did not affect platelet activation induced by a low agonist concentration. Similarly, to investigate whether preincubation of platelets with LPS_SM_ affects agonist-induced platelet responses, PRP was preincubated with LPS_SM_ for 5 minutes at room temperature prior to activation with 0.5 μg/mL CRP-XL but again this did not affect platelet function (Fig. S3C,D).

Many previous studies reporting the ability of LPS to modulate platelet function were conducted at room temperature^13,15,18,29^. To make the experiments more physiologically relevant, the platelets were incubated at 37 °C. This enabled the investigation of whether temperature was a factor conferring the modulation of platelet activation by LPS_SM_. The data obtained with platelets that were preincubated at 37 °C for 5 minutes with LPS_SM_ followed by 20 minutes of stimulation with 0.5 μg/mL CRP-XL at 37 °C suggest that LPS_SM_ did not alter platelet activity (Fig. 4C,D) under these conditions. To determine the impact of incubation time on LPS_SM_-induced effects, the preincubation times were increased from 5 minutes to 30 minutes. The increase in preincubation time did not enable LPS_SM_ to significantly affect fibrinogen binding (Fig. 4E) although, P-selectin exposure stimulated by 0.5 μg/mL CRP-XL was significantly reduced (by approximately 5%) only at a concentration of 2 μg/mL LPS_SM_ (Fig. 4F). At higher concentrations of LPS_SM_, with 10 minutes of pre-incubation at 37 °C, LPS_SM_ was unable to influence fibrinogen binding or P-selectin exposure induced by CRP-XL (Fig. S3E,F). However, under these conditions, 5 μg/mL LPS_SM_ was able to significantly potentiate (by around 30%) the P-selectin exposure induced by 10 μM TRAP-6 but fibrinogen binding was unaffected (Fig. S3G,H). Moreover, ADP-induced fibrinogen binding and P-selectin exposure were unaffected by LPS_SM_ (Fig. S3I,J). Furthermore, whole human blood was used to investigate the effect of LPS_SM_ on platelet activation. The whole human blood was incubated with LPS_SM_ for 5 minutes at 37 °C prior to platelet activation by 0.5 μg/mL CRP-XL for 20 minutes at 37 °C but it did not affect platelet activation in whole blood (Fig. 4G,H). In conclusion, while 2 μg/mL LPS_SM_ is capable of significantly inhibiting (only by around 5%) CRP-XL-induced P-selectin exposure on the platelet surface under specific conditions (PRP; 30 minutes of preincubation at 37 °C), in general, LPS_SM_ did not affect platelet function under any of the conditions tested in this study.

### LPS_RS_ inhibits platelet activation at specific conditions

**Figure 5:**
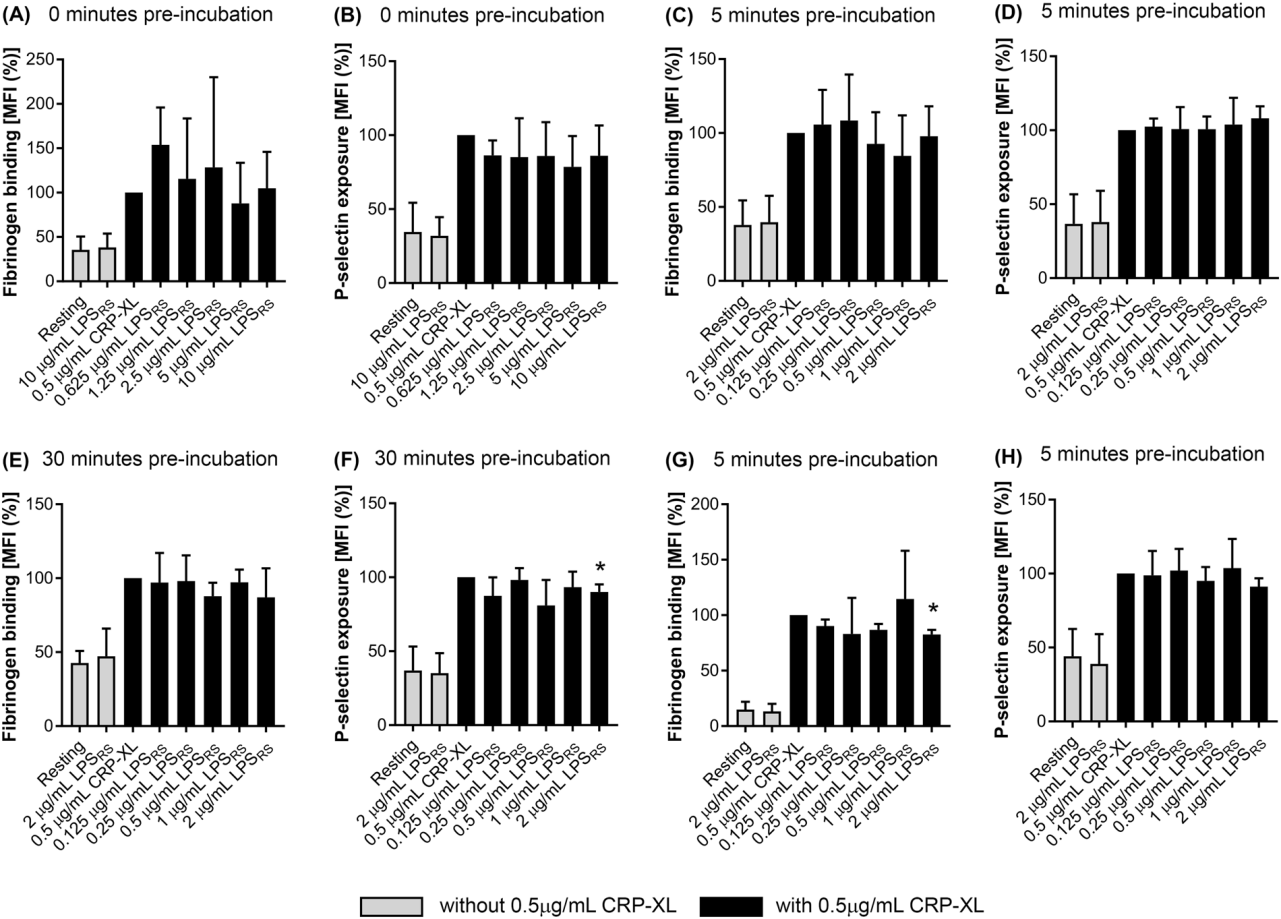
Effect of LPS_RS_ on platelet activation. To determine the impact of LPS_RS_ on the modulation of platelet activation, the level of fibrinogen binding (**A**) and P-selectin exposure (**B**) was analysed in PRP in the presence of different concentrations of LPS_RS_ and 0.5 μg/mL CRP-XL after incubation for 20 minutes at room temperature (n = 4). To determine the impact of temperature on LPS-mediated effects in platelets, the level of fibrinogen binding (**C**) and P-selectin exposure (**D**) in PRP was measured upon preincubation with LPS_RS_ for 5 minutes followed by stimulation with 0.5 μg/mL CRP-XL for 20 minutes at 37 °C (n = 6). Similarly, the preincubation of PRP with LPS_RS_ was increased to 30 minutes prior to stimulation with 0.5 μg/mL CRP-XL for 20 minutes at 37 °C, and the level of fibrinogen binding (**E**) and P-selectin exposure (**F**) was analysed (n = 5). Furthermore, the level of fibrinogen binding (**G**) and P-selectin exposure (**H**) was analysed in human whole blood upon preincubation with LPS_RS_ for 5 minutes followed by stimulation with 0.5 μg/mL CRP-XL for 20 minutes at 37 °C (n = 3). The data were normalised to their 0.5 μg/mL CRP-XL control, and the *p* value (*p ≤ 0.05) shown is as calculated using one-way ANOVA and Dunnett’s post-hoc test. Data represent mean ± S.D. The samples treated in the absence of 0.5 μg/mL CRP-XL are represented with empty bars.

LPS_RS_ has been suggested to be an antagonist for TLR4^13,22,23^. Here, the effect of ultrapure LPS_RS_ was investigated upon activation of platelets with CRP-XL by measuring the level of fibrinogen binding and P-selectin exposure using flow cytometry. LPS_RS_ was tested in the absence of a TLR4 agonist to investigate potential endogenous activity of the receptor as previous work has suggested that some TLR4 molecules are in an active conformation in unstimulated cells^23^. Furthermore, endogenous ligands, such as high mobility group box 1 (HMGB1), have been suggested to activate TLR4^31,32^. Similar to LPS_SM_, LPS_RS_ was tested in resting platelets in all the conditions used in the above experiments (Fig. 5A–H) and the results demonstrate that LPS_RS_ is unable to significantly alter platelet activation in resting platelets.

To determine the impact of LPS_RS_ on agonist-induced platelet activation, platelets were incubated simultaneously with LPS_RS_ and 0.5 μg/mL CRP-XL for 20 minutes at room temperature (Fig. 5A,B) and the levels of fibrinogen binding and P-selectin exposure were measured. The results show that LPS_RS_ did not significantly inhibit P-selectin exposure or fibrinogen binding under these conditions. LPS_RS_ was also tested in conjunction with the lower concentration of CRP-XL (0.25 μg/mL) under the same conditions (Fig. S4A,B). However, it was unable to significantly inhibit platelet activation at this concentration of CRP-XL. Platelets were also preincubated with LPS_RS_ at room temperature for 5 minutes (Fig. S4C,D) before stimulation with CRP-XL (0.5 μg/mL) but it did not alter platelet activation under these conditions. The influence of LPS_RS_ was also tested at 37 °C (Fig. 5C,D) with 5 minutes of preincubation with LPS_RS_ followed by 20 minutes stimulation with 0.5 μg/mL CRP-XL and it did not affect platelet activation. When the preincubation time was increased to 30 minutes, although the level of fibrinogen binding was unaffected under these conditions (Fig. 5E), a significant decrease (approximately 10%) in P-selectin exposure induced by 0.5 μg/mL CRP-XL (Fig. 5F) was observed. The effect of LPS_RS_ on platelet activation in whole blood was examined. Although a decrease in fibrinogen binding was observed (approximately 20%, Fig. 5G), no significant inhibition of P-selectin exposure (Fig. 5H) was observed following 5 minutes of preincubation with LPS_RS_ and 20 minutes of stimulation with CRP-XL at 37 °C. Together, these data demonstrate that ultrapure LPS_RS_ is able to significantly inhibit P-selectin exposure (α-granule secretion in PRP) following 30 minutes preincubation, and inside-out signalling to integrin α_IIb_β_3_ in whole blood, but in other conditions, LPS_RS_ does not alter platelet activation either directly or upon activation with a platelet agonist.

Moreover, following 10 minutes of preincubation with ultrapure LPS_RS_, platelet fibrinogen binding and P-selectin exposure induced by 0.5 μg/mL CRP-XL (S4E-F), 10 μM TRAP-6 (S4G-H), and 10 μM ADP (S4I-J) were unaffected by the presence of ultrapure LPS_RS_. Interestingly, when these experiments were repeated using a non-ultrapure version of LPS_RS,_ fibrinogen binding (but not P-selectin exposure) induced by 0.5 μg/mL CRP-XL was potentiated by 5 μg/mL LPS_RS_ (Fig. S4K,L). Furthermore, no significant change in responses evoked by 10 μM TRAP-6 was observed in fibrinogen binding and P-selectin exposure (Fig. S4M,N). However, a significant increase was observed in P-selectin exposure (but not fibrinogen binding) in platelets induced by 10 μM ADP in the presence of 10 μg/mL LPS_RS_ (Fig. S4O,P). This provides further evidence that there may be a contaminant present in these non-ultrapure versions of LPS that may be responsible for stimulating platelet activity.

### LPS does not affect aggregation of pre-activated platelets

**Figure 6:**
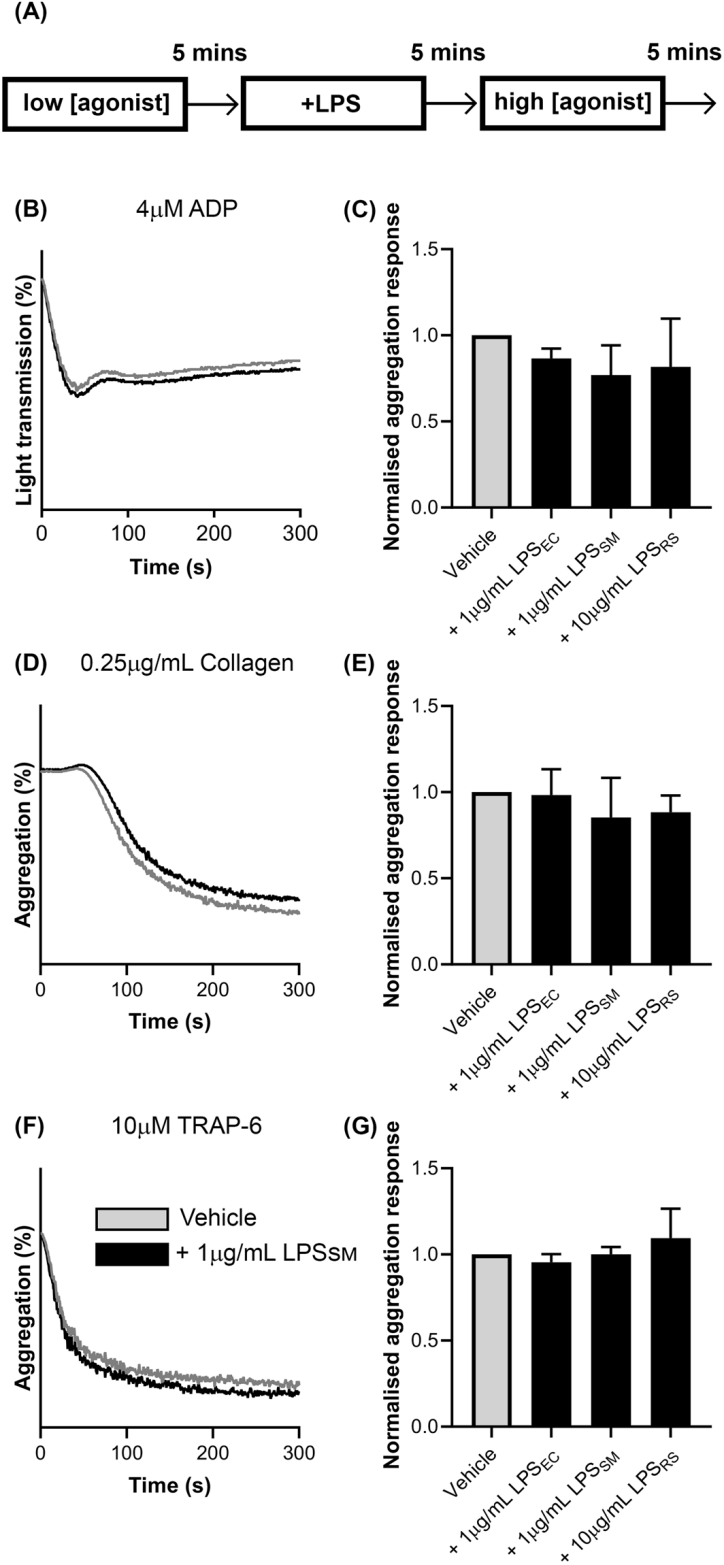
Effect of pre-activation of platelets on the LPS-mediated actions. Aggregometry was used to determine the effect of mild pre-activation by a low dose of different platelet agonists on LPS-modulated actions in PRP. (**A**) demonstrates the schematic protocol for this experiment. A representative trace demonstrating the aggregation induced by ADP in the presence of a vehicle control or 1 μg/mL LPS_SM_ is shown in (**B**). The extent of activation by 4 μM ADP following pre-activation with 0.5 μM ADP and treatment with vehicle, 1 μg/mL LPS_EC_, 1 μg/mL LPS_SM_, or 10 μg/mL LPS_RS_ is shown in (**C**). (**D**) A representative aggregation curve induced by 0.25 μg/mL collagen in PRP following pre-activation by 0.1 μg/mL and incubation with a vehicle control or 1 μg/mL LPS_SM_. (**E**) Shows the normalised results for this experiment. (**F**) A representative trace of aggregation induced by 10 μM TRAP-6 following pre-activation with 1 μM TRAP-6 and incubation with a vehicle control or 1 μg/mL LPS_SM_. The normalised aggregation response for this experiment is shown in (**G**). In all traces, the LPS-treated trace is represented in black whilst the vehicle is represented in grey. Data represent mean ± S.D. (n = 3) and were analysed using one-way ANOVA.

As the availability of TLR4 on the platelet surface has been suggested to increase upon platelet activation^11,26^, platelets were pre-activated with a low concentration of ADP (0.5 μM) prior to treatment with LPS_EC_, LPS_SM_, or LPS_RS_ and further activation with a higher dose of ADP (4 μM) (Fig. 6A). The results (Fig. 6B,C) suggest that none of the three ultrapure LPS chemotypes are capable of significantly modifying the aggregation of platelets under these conditions. Similar results were obtained when the experiment was conducted, using the same procedure, with 0.1 μg/mL collagen (low dose) and 0.25 μg/mL collagen (high dose; Fig. 6D,E) or 1 μM TRAP-6 (low dose) and 10 μM TRAP-6 (high dose; Fig. 6F,G). These data suggest that priming platelets did not affect the ability of LPS chemotypes to modulate platelet function.

### LPS does not exert cytotoxic effects in platelets

To determine if the ultrapure LPS chemotypes used in this study exert toxic effects in platelets, various concentrations of LPS chemotypes were analysed in platelets using a lactate dehydrogenase (LDH) assay. The results suggest that the LPS chemotypes [LPS_EC_ (Fig. S5A), LPS_SM_ (Fig. S5B), and LPS_RS_ (Fig. S5C)] at the concentrations used in this study were unable to induce cytotoxicity in platelets. The results demonstrate that ultrapure LPS chemotypes do not induce any direct cytotoxic effects on platelets.

## Discussion

Several studies have reported controversial results on the effects of LPS (specifically LPS_EC_) on platelet function. A key source of criticism revolves around the potential contamination of LPS preparations, possibly by cell wall components that may stimulate TLR2, and the interference of these contaminants on platelet reactivity^20^. The structure of LPS varies between chemotypes and these structural differences have been proposed to be responsible for conferring different downstream activities^10,11,33^. In order to address some of these previous concerns and to determine the precise actions of LPS on platelets, we determined the impact of ultrapure LPS chemotypes from various bacterial species on the modulation of platelet activation.

Initially, the activity of LPS chemotypes was analysed using an NF-κB reporter cell line (which selectively express TLR4 and lack TLR2) to confirm their ability to selectivity bind TLR4 and induce downstream signalling. Our results demonstrate that both ultrapure LPS_EC_ and LPS_SM_ are capable of increasing luciferase activity in U251-NF-κB-GFP-Luc cells compared to the controls whereas LPS_RS_ did not affect the activity as it is an antagonist for TLR4^23^. Moreover, LPS_EC_ and LPS_SM_ were incapable of significantly inducing signalling to NFκB in the presence of LPS_RS_. Similar results were reported in a previous study to demonstrate the impact of ultrapure LPS chemotypes in the modulation of luciferase activity in the same cell type^24^.

The expression of TLR4 on the platelet surface has been widely reported and indeed, in some studies this has been reported to increase upon activation of platelets by a diverse range of agonists including thrombin, convulxin, TRAP-6, ADP, and adrenaline^11,16,26^. Here, we also demonstrate that platelets express low levels of TLR4 on the surface at resting conditions, however its level increases following activation with agonists such as CRP-XL and collagen. Moreover, Tsai *et al*.^26^ suggested that in resting platelets, TLR4 is associated with myosin-9 in the intracellular α-granules, and during activation by thrombin, calpain (a protease) is activated and it cleaves the interactions between these two proteins and liberates TLR4 enabling its transport to the platelet surface. Consistent with this previous study, we demonstrate that TLR4 (at 94 kDa) was only prominently detectable in activated platelets. The resting platelets may contain TLR4, however, if it is associated with myosin-9 in α-granules, it may possess a greater molecular mass. Thus, it is not expected to appear at 94 kDa. Furthermore, this would suggest that the preincubation of TLR ligands is superfluous as there may not be a substantial number of TLR4 receptors present on the platelet surface. Moreover, the level of platelet activation by agonists could be a key factor for TLR4 exposure on the cell surface. However, we demonstrate that LPS_RS_ and LPS_SM_ treatments required 30 minutes of preincubation at 37 °C before a significant (~10%) decrease in P-selectin exposure was observed at a supraphysiological concentration under specific conditions.

Previously, it has been reported that LPS_EC_ can enhance platelet aggregation^13,18^ and alter the release of different cytokines^11,29^. Furthermore, NF-ĸB (the transcription factor activated downstream of the MyD88-dependent pathway) is involved in platelet activation induced by classical platelet agonists^2,6,34^. Notably, platelets obtained from TLR4-deficient mice have been shown to possess similar aggregation behaviour compared to control mouse platelets^35^. Currently, there is no consensus regarding platelet response to LPS_EC_ as some reports suggest that it can induce P-selectin exposure and fibrinogen binding^7,13,18,30^ whereas others suggest that it does not^11,15,17^. Ultrapure LPS_EC_ has been used by Berthet *et al*.^11^ and Claushuis *et al*.^15^ where there was no significant increase in P-selectin exposure observed. In line with these previous studies, here we also report that LPS_EC_ does not modulate platelet activation under the diverse settings used in this study. However, a non-ultrapure version of LPS_EC_ did induce a significant increase in the binding of fibrinogen to platelets which supports the hypothesis that the contaminants found in LPS preparation may be responsible for inducing the observed effects in platelets. The incubation times and concentrations of LPS_EC_ (0.125 μg/mL and 2 μg/mL) used in this study are comparable to other studies where they have used concentrations ranging from 100 ng/mL^15^ to 500 ng/mL^29^, 1 μg/mL^13,18,36^ to 3 μg/mL^11^, and 5 μg/mL^3,7^ to 10 μg/mL^30^.

Moreover, LPS_SM_ did not affect fibrinogen binding and P-selectin exposure in the absence of a platelet agonist. The preincubation (30 minutes) of LPS_SM_ with platelets was able to significantly reduce agonist-induced P-selectin exposure (but only by around 5%) suggesting that α-granule secretion may be inhibited at supraphysiological concentrations of LPS_SM_. Moreover, the level of fibrinogen binding was unaffected by LPS_SM_ under any of the conditions tested.

LPS_RS_ was also tested for its effects on platelet activity under similar conditions to LPS_SM_ but it did not display any significant effects in the absence of a platelet agonist. However, it affected α-granule secretion but only at supraphysiological concentrations after 30 minutes of preincubation in the PRP. Conversely, the non-ultrapure LPS_RS_ significantly increased fibrinogen binding to platelets upon activation with CRP-XL and P-selectin exposure on platelets upon activation with ADP, which suggests that another factor may be present in the non-ultrapure version that induces the effects reported here and in other studies. To investigate whether pre-activation of platelets would augment TLR4-mediated effects, a low dose of agonist (ADP, TRAP-6, or collagen) was used to prime platelets prior to LPS treatment and further stimulation with a greater concentration of the same agonist. These experiments did not lead to an increase in the effect induced by any of the LPS chemotypes used.

Under pathological conditions such as in sepsis, a plethora of factors are present in the bloodstream and they may be responsible for the observed *in vivo* effects of LPS on platelets. This could be explained by synergistic effects that were not tested in this study although this was suggested by other studies^15,19,37^. In addition to platelets, many other immune cells present in the blood may also respond to LPS and interact with platelets thereby augmenting their activity^1,3,38^. Despite the presence of immune cells in the whole blood, the results from this study suggest that the LPS_EC_ and LPS_SM_ treatment is unable to alter fibrinogen binding or P-selectin exposure on platelets in whole blood. However, LPS_RS_ was capable of modulating inside-out signalling to integrin α_IIb_β_3_, but not α-granule secretion following stimulation with an agonist at specific concentrations.

Overall, we conclude that the actions of LPS chemotypes on platelets may not be direct and, during pathological conditions, this may be driven or augmented through other molecules that are released under those circumstances. Moreover, the purity of LPS must be ensured prior to testing them in platelets either through a reporter cell line or other experiments to confirm their selectivity to TLR4 and the absence of potential impurities for other molecules. It is important that physiological effects mediated via other receptors, such as TLR2, are not misinterpreted as TLR4-specific effects. This will ensure that solid foundations are available for clinical research, as TLR4 remains an attractive target for certain immunological diseases. The results presented in this study will form a strong basis for future studies to investigate the impact of different LPS chemotypes on the modulation of platelet function, haemostasis, and thrombosis under diverse pathophysiological settings.

## Methods

### Materials

Ultrapure lipopolysaccharides (LPS) from *Escherichia coli* O111:B4, *Salmonella minnesota* R595, and *Rhodobacter sphaeroides* and their non-ultrapure alternatives (where available) were purchased from InvivoGen, France and used throughout all the experiments. Ultrapure versions of LPS were purified by an additional phenol-TEA-DOC step compared to the non-ultrapure versions that underwent extraction via a phenol-water mixture^21^.

### Human blood collection and platelet preparation

The blood was obtained from healthy human volunteers with informed consent in accordance to the procedures approved by the University of Reading Research Ethics Committee (UREC: 17/17), and the platelets were prepared as described previously^39–42^. All methods were performed in accordance with the relevant institutional and national guidelines and regulations. Briefly, the blood was drawn via venepuncture into vacutainers containing 3.2% (w/v) citrate and used in assays where whole blood was required. For the preparation of platelet-rich plasma (PRP), the blood was centrifuged at 102 *g* for 20 minutes at 20 °C and the PRP was carefully collected for further experiments. For the preparation of isolated platelets, the blood was mixed with 15% (v/v) acid citrate dextrose [ACD: 2.5% (w/v) sodium citrate, 2% (w/v) glucose and 1.5% (w/v) citric acid] prior to centrifugation at 102 *g* for 20 minutes at 20 °C. The PRP was then collected and centrifuged at 1413 *g* for 10 minutes at 20 °C in the presence of 50 ng/mL prostaglandin I_2_ (PGI_2_) before the plasma was removed and the platelet pellet was resuspended in modified Tyrode’s-HEPES buffer (134 mM NaCl, 2.9 mM KCl, 0.34 mM Na_2_HPO_4_.12H_2_O, 12 mM NaHCO_3_, 20 mM HEPES, 1 mM MgCl_2_, and 5 mM D-glucose, pH 7.3) with 12% (v/v) ACD. Following another centrifugation at 1413 *g* for 10 minutes in the presence of 50 ng/mL PGI_2_, the platelet pellet was resuspended in fresh modified Tyrode’s-HEPES buffer and left to rest for 30 minutes at 30 °C prior to use.

### Cell culture and luciferase assay

The U251-NF-κB-GFP-Luc cells^24^ grown in high glucose DMEM supplemented with 10% (v/v) foetal calf serum (FCS) and 2 mM L-glutamine (Sigma Aldrich, UK) were seeded on a 24-well plate at 1 × 10^5^ cells/well and left in a 37 °C humidified incubator with 5% CO_2_ until >80% confluency was achieved. The cells were then starved in serum-free high glucose DMEM supplemented with 2 mM L-glutamine for 3.5 hours prior to addition of vehicle (endotoxin-free water) or 10 μg/mL LPS_RS_ for 30 minutes. Subsequently, cells were treated with a vehicle control, 1 μg/mL LPS_EC_, 1 μg/mL LPS_SM_, or 10 μg/mL LPS_RS_ (or their combinations). After 24 hours, the cells were washed with sterile phosphate-buffered saline (PBS) (Sigma Aldrich, UK) before being lysed on a rocker in cell culture lysis buffer (Promega, UK) for one hour at room temperature. The cell lysates were centrifuged for 5 minutes at 5000 *g* at room temperature. The level of luciferase activity of each lysate after the addition of luciferase assay substrate (Promega, UK) was measured by a SpectraMax iD3 multi-mode microplate reader (Molecular Devices, USA).

### Immunocytochemistry

The isolated human platelets were suspended in modified Tyrode’s-HEPES buffer containing 10 μM Cell Tracker, CMAC (Thermo Fisher Scientific, UK) for one hour. The platelets were mixed on a rotational plate shaker at 330 rpm for 10 minutes at room temperature in the presence of 0.5 μg/mL cross-linked collagen-related peptide (CRP-XL) (obtained from Professor Richard Farndale, University of Cambridge, UK) or modified Tyrode’s buffer as a control before they were fixed by the addition of 0.2% (v/v) formyl saline. The platelets were centrifuged at 2500 *g* for 5 minutes, washed in PBS, then resuspended in 5% goat serum in PBS for blocking for 30 minutes. After blocking, the platelets were washed with PBS and resuspended in 1/100 anti-TLR4 [76B357.1] antibody (Abcam, UK) in PBS and incubated for one hour prior to washing and incubating with 1/300 goat anti-mouse Alexa Fluor 647-conjugated secondary antibody for one hour. Subsequently, the platelets were washed, resuspended in Mowiol [containing 0.1% (v/v) 1,4-phenylenediamine dihydrochloride], and mounted on a microscope slide. A Nikon A1-R Confocal Microscope was used for image acquisition using a 100x objective. The level of expression of TLR4 was quantified using ImageJ.

### Immunoblotting

The isolated human platelets were treated with either modified Tyrode’s-HEPES buffer (resting control) or 0.5 μg/mL CRP-XL for five minutes in an aggregometer prior to lysis in reducing sample treatment buffer [RSTB; 69 mM sodium dodecyl sulphate, 5% (v/v) 2-mercaptoethanol, 10% (v/v) glycerol, and 25 mM Tris-HCl]. Subsequently, the platelet lysates were boiled at 90 °C for 10 minutes and analysed by SDS-PAGE in 4–15% pre-cast gels (Bio-Rad, UK) and then transferred to a PVDF membrane (GE Healthcare, UK) using a Semi-Dry Transfer System (Bio-Rad, UK). The membrane was blocked with 5% (w/v) bovine serum albumin (BSA) in PBS with 0.1% (v/v) Tween-20 (PBS-T) for 1 hour at room temperature. Then it was incubated overnight at 4 °C with anti-TLR4 antibody (1/250 dilution) and for 1 hour at room temperature with anti-α-tubulin [B-7] or anti-14-3-3ζ antibody (1/2000 dilution) (Santa Cruz Biotechnology, USA). 30 μg of HEK-293 or U251-NF-κB-GFP-Luc cell lysates were used as a TLR4-negative or -positive control respectively. The primary antibodies were detected by using Cy5-conjugated goat anti-mouse IgG (1/2500 dilution) (Thermo Fisher Scientific, UK) in a Typhoon 9400 variable mode imager (GE Healthcare, UK) (488 V) and images were analysed using ImageJ.

### Flow cytometry-based assays

Rabbit polyclonal FITC-conjugated anti-human fibrinogen antibodies (Dako, UK) were used to measure the level of fibrinogen binding as a marker for inside-out signalling to integrin α_IIb_β_3_^28^ and PE-Cy5-conjugated mouse anti-human CD62P antibodies (BD Biosciences, UK) were used to measure the level of P-selectin exposure as a marker for α-granule secretion from platelets^1,29^. The human PRP was incubated with both the antibodies in HEPES-buffered saline (HBS: 150 mM NaCl, 5 mM KCl, 2 mM MgSO_4_.7H_2_O, and 10 mM HEPES, pH 7.4) for various time periods (0, 5, 20, 25, 30 and 50 minutes) with and without different concentrations of LPS chemotypes (LPS_EC_, LPS_SM_, and LPS_RS_). After preincubation, the platelets were exposed to modified Tyrode’s-HEPES buffer (vehicle) or 0.5 μg/mL CRP-XL, 0.25 μg/mL CRP-XL, 10 μM TRAP-6 (Abcam, UK), or 10 μM ADP (Sigma, UK) for various time points at room temperature or 37 °C. The platelets were then fixed using 0.2% (v/v) formyl saline and the level of fluorescence was measured using an Accuri C6 Flow cytometer (BD Biosciences, UK).

### Platelet aggregation

The PRP was pre-activated with 0.5 μM ADP, 1 μM TRAP-6, or 0.1 μg/mL collagen (ChronoLog, UK) for five minutes prior to the addition of different ultrapure LPS chemotypes and incubation for another five minutes before initiating aggregation with 2 μM ADP, 10 μM TRAP-6, or 0.25 μg/mL collagen respectively. The aggregation was monitored for five minutes using a Chrono-Log (Model 700) aggregometer (USA) under constant stirring conditions at 37 °C.

### Lactate dehydrogenase (LDH) assay

In order to determine if the LPS chemotypes have direct cytotoxic effects, a LDH cytotoxicity assay was performed using a LDH Cytotoxicity Assay Kit (Thermo Fisher Scientific, UK) according to the manufacturer’s instructions. Briefly, human PRP was incubated at 37 °C for 30 minutes prior to incubation with different concentrations of LPS chemotypes or a vehicle control (endotoxin free water) for 5 minutes. Subsequently, the reaction mixture provided in the kit was added to platelets and incubated for 30 minutes and the reaction was stopped using a stop solution (provided in the kit). The positive control referred to in the results was supplied in the kit. The absorbance of the samples was read at 490 nm and 650 nm using a Fluostar Optima (BMG Labtech, Germany) spectrofluorometer.

### Statistical analysis

All the data obtained in this study were analysed using GraphPad Prism 8. The data obtained from cell culture experiments were analysed using one-way ANOVA with the differences between treatments investigated using a Bonferroni’s post-hoc test. Comparisons for relative band intensity in immunoblot images and TLR4 cluster:platelet ratios were analysed via Student’s t-test. Flow cytometry experiments were analysed using one-way ANOVA. The differences between the vehicle control (for experiments involving LPS_EC_) or the positive control (0.25 μg/mL, 0.5 μg/mL CRP-XL, 10 μM TRAP-6, or 10 μM ADP) and the experimental mean were tested for statistical significance through the use of a Dunnett’s post-hoc test. The normality of distribution was examined for all the datasets and non-parametric tests were used where appropriate (Friedman’s test with Dunn’s post-hoc test).

## Supplementary information

Supplementary Information

## Data Availability

The datasets generated and analysed in this study are available from the corresponding author on reasonable request.

## References

[1] Von Hundelshausen P, Weber C (2007). Platelets as immune cells: Bridging inflammation and cardiovascular disease. Circ. Res..

[2] Vallance TM, Zeuner M, Williams HF, Widera D, Vaiyapuri S (2017). Toll-Like Receptor 4 Signalling and Its Impact on Platelet Function, Thrombosis, and Haemostasis. Mediators Inflamm..

[3] Clark SR (2007). Platelet TLR4 activates neutrophil extracellular traps to ensnare bacteria in septic blood. Nat. Med..

[4] Youssefian T, Drouin A, Massé JM, Guichard J, Cramer EM (2002). Host defense role of platelets: Engulfment of HIV and Staphylococcus aureus occurs in a specific subcellular compartment and is enhanced by platelet activation. Blood.

[5] Cognasse F (2015). The inflammatory role of platelets via their TLRs and Siglec receptors. Front. Immunol..

[6] Zeuner MT (2016). Biased signalling is an essential feature of TLR4 in glioma cells. Biochim. Biophys. Acta.

[7] Andonegui G (2005). Platelets express functional Toll-like receptor-4 (TLR4). Blood.

[8] Berthet J (2010). Toll-like receptor 4 signal transduction in platelets: novel pathways. Br. J. Haematol..

[9] Karim ZA, Vemana HP, Khasawneh FT (2015). MALT1-ubiquitination triggers non-genomic NF-κB/IKK signaling upon platelet activation. PLoS One.

[10] Panzer S (2013). Differential response to LPS isotypes induced platelet activation mediated by Toll-like receptor (TLR)-4. Clin. Immunol..

[11] Berthet J (2012). Human platelets can discriminate between various bacterial LPS isoforms via TLR4 signaling and differential cytokine secretion. Clin. Immunol..

[12] Kappelmayer J (2013). Distinct effects of Re- and S-forms of LPS on modulating platelet activation. J. Thromb. Haemost..

[13] Lopes Pires ME, Clarke SR, Marcondes S, Gibbins JM (2017). Lipopolysaccharide potentiates platelet responses via toll-like receptor 4-stimulated Akt-Erk-PLA2signalling. PLoS One.

[14] Damien P (2015). LPS stimulation of purified human platelets is partly dependent on plasma soluble CD14 to secrete their main secreted product, soluble-CD40-Ligand. BMC Immunol..

[15] Claushuis Theodora A.M., Van Der Veen Annelou I.P., Horn Janneke, Schultz Marcus J., Houtkooper Riekelt H., Van ’T Veer Cornelis, Van Der Poll Tom (2018). Platelet Toll-like receptor expression and activation induced by lipopolysaccharide and sepsis. Platelets.

[16] Koessler J (2019). The Role of Human Platelet Preparation for Toll-Like Receptors 2 and 4 Related Platelet Responsiveness. TH Open.

[17] Ward JR (2005). Agonists of Toll-like receptor (TLR)2 and TLR4 are unable to modulate platelet activation by adenosine diphosphate and platelet activating factor. Thromb. Haemost..

[18] Zhang G (2009). LPS stimulates platelet secretion and potentiates platelet aggregation via TLR4/MyD88 and the cGMP-dependent protein kinase pathway. J. Immunol..

[19] Aslam R (2006). Platelet Toll-like receptor expression modulates lipopolysaccharide-induced thrombocytopenia and tumor necrosis factor- alpha production *in vivo*. Blood.

[20] Zeuner M, Bieback K, Widera D (2015). Controversial role of Toll-like receptor 4 in adult stem cells. Stem Cell Rev. Reports.

[21] Hirschfeld M, Ma Y, Weis JH, Vogel SN, Weis JJ (2000). Cutting Edge: Repurification of Lipopolysaccharide Eliminates Signaling Through Both Human and Murine Toll-Like Receptor 2. J. Immunol..

[22] Shashkin PN, Brown GT, Ghosh A, Marathe GK, McIntyre TM (2008). Lipopolysaccharide is a direct agonist for platelet RNA splicing. J. Immunol..

[23] Krüger CL, Zeuner M-T, Cottrell GS, Widera D, Heilemann M (2017). Quantitative single-molecule imaging of TLR4 reveals ligand-specific receptor dimerization. Sci. Signal..

[24] Zeuner M, Vallance T, Vaiyapuri S, Cottrell GS, Widera D (2017). Development and characterisation of a novel NF-κB reporter cell line for investigation of neuroinflammation. Mediators Inflamm..

[25] Cognasse F (2005). Evidence of Toll-like receptor molecules on human platelets. Immunol. Cell Biol..

[26] Tsai JC (2014). The role of calpain-myosin 9-Rab7b pathway in mediating the expression of toll-like receptor 4 in platelets: A novel mechanism involved in α-granules trafficking. PLoS One.

[27] Smethurst PA (2007). Structural basis for the platelet-collagen interaction: The smallest motif within collagen that recognizes and activates platelet Glycoprotein VI contains two glycine-proline-hydroxyproline triplets. J. Biol. Chem..

[28] Watson SP, Auger JM, McCarty OJT, Pearce AC (2005). GPVI and integrin alphaIIb beta3 signaling in platelets. J. Thromb. Haemost..

[29] Cognasse F (2008). Toll-like receptor 4 ligand can differentially modulate the release of cytokines by human platelets. Br. J. Haematol..

[30] Rivadeneyra L (2014). Regulation of platelet responses triggered by Toll-like receptor 2 and 4 ligands is another non-genomic role of nuclear factor-kappaB. Thromb. Res..

[31] Vogel S (2015). Platelet-derived HMGB1 is a critical mediator of thrombosis. J Clin Invest.

[32] Yang X (2015). HMGB1: a novel protein that induced platelets active and aggregation via Toll-like receptor-4, NF-κB and cGMP dependent mechanisms. Diagn. Pathol..

[33] Billod J-M, Lacetera A, Guzmán-Caldentey J, Martín-Santamaría S (2016). Computational approaches to Toll-Like receptor 4 modulation. Molecules.

[34] Malaver E (2009). NF-κB inhibitors impair platelet activation responses. J. Thromb. Haemost..

[35] Stark RJ, Aghakasiri N, Rumbaut RE (2012). Platelet-derived Toll-like receptor 4 (TLR-4) is sufficient to promote microvascular thrombosis in endotoxemia. PLoS One.

[36] Assinger A, Laky M, Badrnya S, Esfandeyari A, Volf I (2012). Periodontopathogens induce expression of CD40L on human platelets via TLR2 and TLR4. Thromb. Res..

[37] de Stoppelaar SF (2015). The role of platelet MyD88 in host response during gram-negative sepsis. J. Thromb. Haemost..

[38] Sabroe I, Jones EC, Usher LR, Whyte MKB, Dower SK (2002). Toll-Like Receptor (TLR)2 and TLR4 in Human Peripheral Blood Granulocytes: A Critical Role for Monocytes in Leukocyte Lipopolysaccharide Responses. J. Immunol..

[39] Vaiyapuri S (2013). Connexin40 regulates platelet function. Nat. Commun..

[40] Vaiyapuri S, Sage T, Rana R, Schenk M (2015). EphB2 regulates contact-dependent and independent signalling to control platelet function. Blood.

[41] Ravishankar D (2017). Ruthenium-conjugated chrysin analogues modulate platelet activity, thrombus formation and haemostasis with enhanced efficacy. Sci. Rep..

[42] Ravishankar D (2018). Impact of specific functional groups in flavonoids on the modulation of platelet activation. Sci. Rep..

