# Supplementary material for: Effect of ultrapure lipopolysaccharides derived from diverse bacterial species on the modulation of platelet activation
Source: Sci Rep. 2019 Dec 3;9:18258. doi: 10.1038/s41598-019-54617-w (PMC6890654; doi:10.1038/s41598-019-54617-w)
Supplement: Supplementary file 1 — Supplementary Information [file 41598_2019_54617_MOESM1_ESM.docx]

**Effect of ultrapure lipopolysaccharides derived from diverse bacterial species on the modulation of platelet activation**

Thomas M. Vallance^1^, Divyashree Ravishankar^1^, Dina A. I. Albadawi^1^, Harry Layfield^1^, Jonathan Sheard^2,3^, Rajendran Vaiyapuri^4^, Philip Dash^5^, Ketan Patel^5^, Darius Widera^2^ and Sakthivel Vaiyapuri^1^*

^1^School of Pharmacy, University of Reading, Reading, RG6 6UB, United Kingdom

^2^Stem Cell Biology and Regenerative Medicine Group, School of Pharmacy, University of Reading, Reading, RG6 6UB, United Kingdom

^3^Sheard BioTech Ltd, 20-22, Wenlock Road, London N1 7GU, United Kingdom

^4^School of Pharmacy, University of Reading Malaysia, Johor, Malaysia

^5^School of Biological Sciences, University of Reading, Reading, RG6 6UB, United Kingdom

*Corresponding author: Dr Sakthivel Vaiyapuri, School of Pharmacy, University of Reading, Reading, RG6 6UB, United Kingdom. Email: [s.vaiyapuri@reading.ac.uk](mailto:s.vaiyapuri@reading.ac.uk)

**Supplementary Information**

**
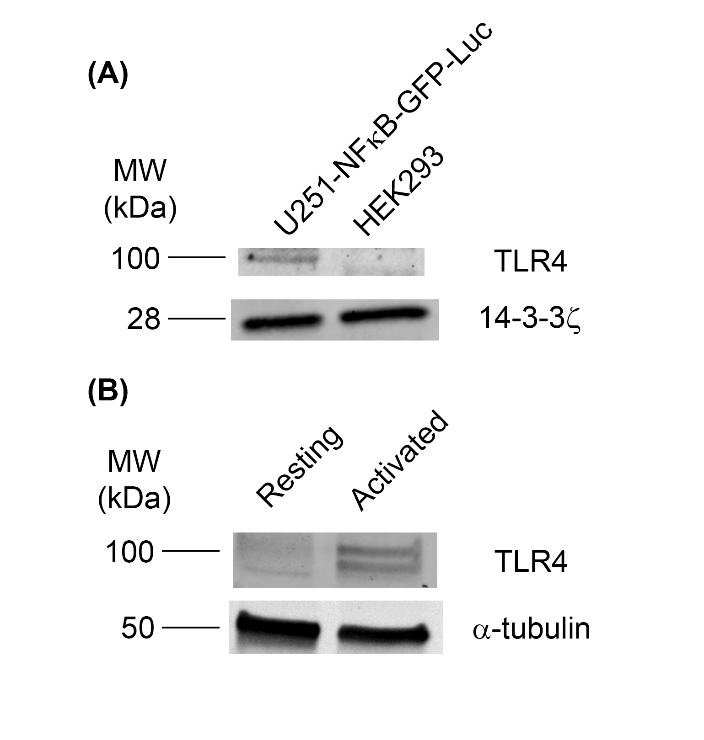
**

Figure S1: **TLR4 expression in platelets and control cells*.* (A)** U251-NF-κB-GFP-Luc cell lysates (left) and HEK293 cell lysates (right) were used as positive and negative controls respectively to validate the anti-TLR4 antibody used. **(B)** Platelet lysates stimulated with 1μg/mL collagen and their resting equivalents were also probed for their TLR4 expression using the anti-TLR4 antibody. The blot shown is representative of three separate experiments.


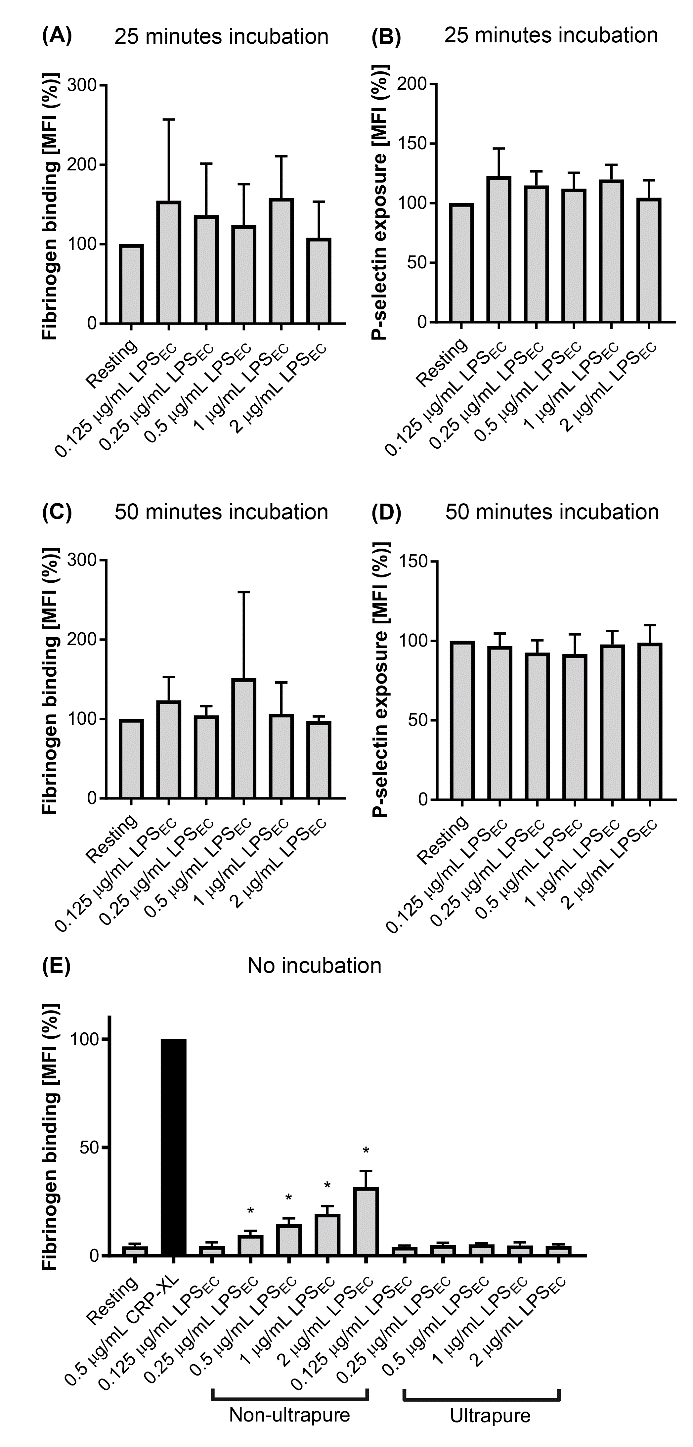


Figure S2: **Effect of LPS_EC_ on platelet activation.** The level of fibrinogen binding and P-selectin exposure in PRP was measured upon incubation with ultrapure LPS_EC_ for 25 minutes at room temperature (**A**, **B**) or 50 minutes at 37°C (n=5) (**C**, **D**). Furthermore, the level of fibrinogen binding to platelets treated with a non-ultrapure version of LPS_EC_ following 20 minutes of incubation was determined (n=3) (**E**). All data were normalised to their resting control and analysed using one-way ANOVA and Dunnett’s post-hoc test (**p*<0.05). Data represent mean ± S.D.


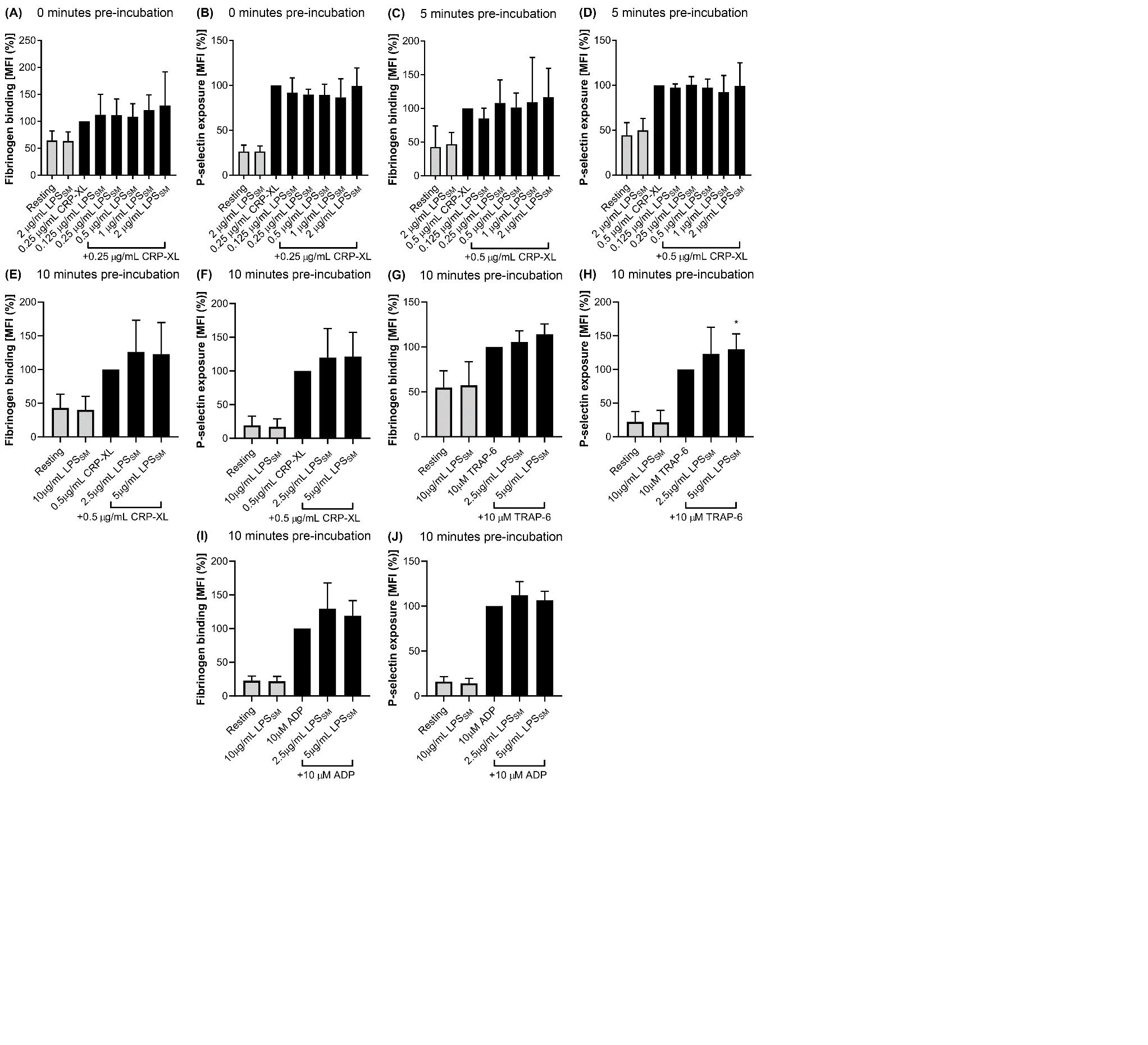


Figure S3: ***Effect of LPS_SM_ on platelet activation***. The level of fibrinogen binding and P-selectin exposure was measured in PRP in the presence of different concentrations of LPS_SM_ and 0.25μg/mL CRP-XL after simultaneous incubation for 20 minutes at room temperature (**A**, **B**) (n=3) or preincubation with LPS_SM_ for 5 minutes followed by stimulation with 0.5μg/mL CRP-XL for 20 minutes at room temperature (n=5) (**C**, **D**). Similarly, higher LPS_SM­_ concentrations were tested with 0.5μg/mL CRP-XL following 10 minutes of pre-incubation and the level of fibrinogen binding (**E**) and P-selectin exposure (**F**) was measured (n=7). This experimental set-up was repeated with 10μM TRAP-6 (**G** and **H**; n=7) and 10μM ADP as platelet agonists (**I** and **J**; n=7). The data were normalised to their 0.5μg/mL CRP-XL, 10μM TRAP-6, or 10μM ADP controls and analysed using one-way ANOVA and Dunnett’s post-hoc test (**p*<0.05). Data represent mean ± S.D.


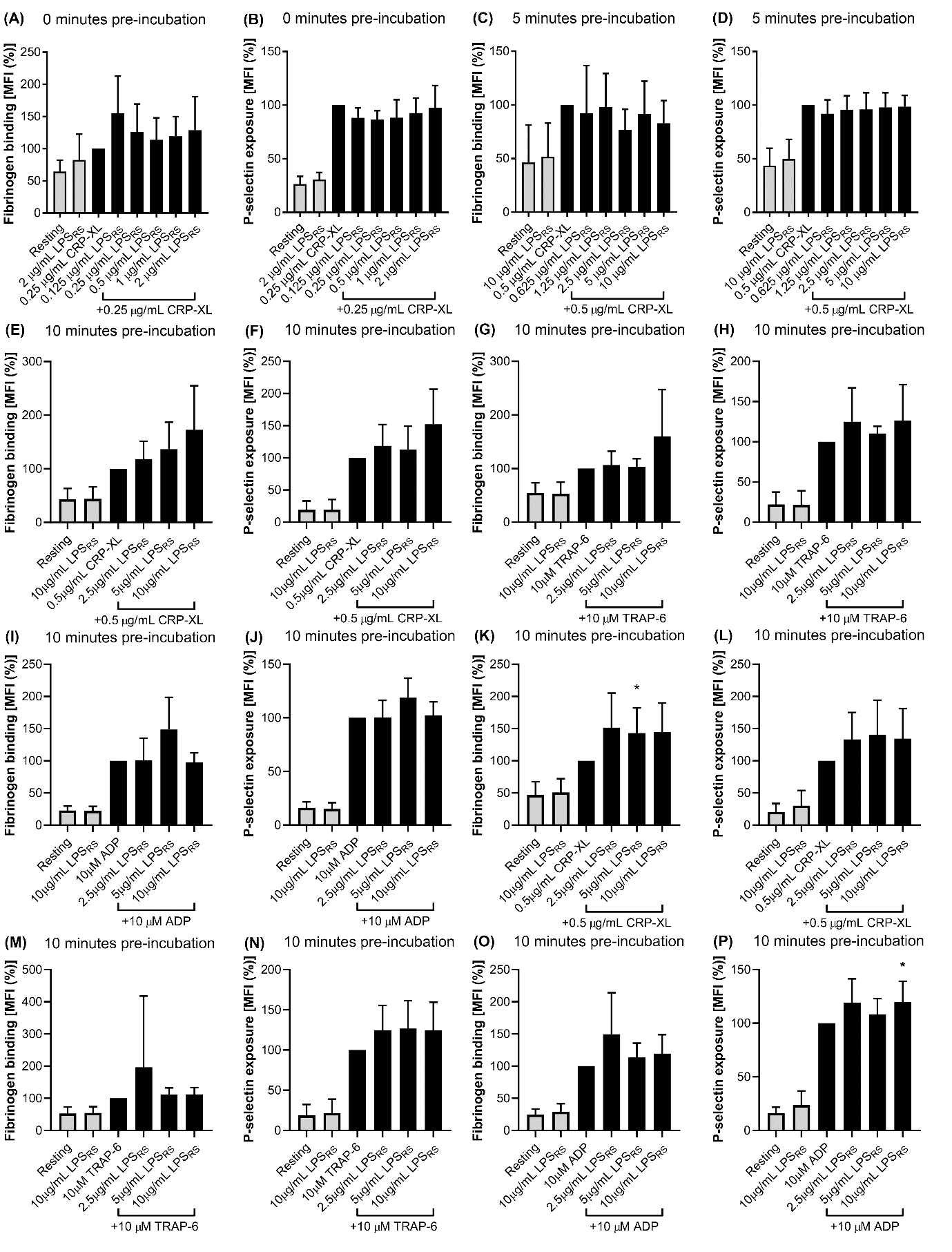


Figure S4: ***Effect of LPS_RS_ on platelet activation.*** The level of fibrinogen binding and P-selectin exposure was measured in PRP in the presence of different concentrations of LPS_RS_ and 0.25μg/mL CRP-XL after incubation for 20 minutes at room temperature (**A**, **B**) (n=4) or preincubation with LPS_RS_ for 5 minutes followed by stimulation with 0.5μg/mL CRP-XL for 20 minutes at room temperature (n=4) (**C**, **D**). Similarly, ultrapure LPS_RS­_ concentrations were tested with 0.5μg/mL CRP-XL following 10 minutes of pre-incubation and the level of fibrinogen binding (**E**) and P-selectin exposure (**F**) was measured (n=7). This experimental set-up was repeated with 10μM TRAP-6 (**G** and **H**; n=7) and 10μM ADP as platelet agonists (**I** and **J**; n=7). Furthermore, non-ultrapure LPS_SM­_ was also tested for its effect on fibrinogen binding and P-selectin exposure in response to 0.5μg/mL CRP-XL (**K** and **L**; n=9), 10μM TRAP-6 (**M** and **N**; n=10), and 10μM ADP (**O** and **P**; n=10) following 10 minutes of pre-incubation. The data were normalised to their 0.5μg/mL CRP-XL, 10μM TRAP-6, or 10μM ADP controls and were analysed using a one-way ANOVA and Dunnett’s post-hoc test (**p*<0.05). Data represent mean ± S.D. The samples treated in the absence of 0.5μg/mL CRP-XL are represented with empty bars.


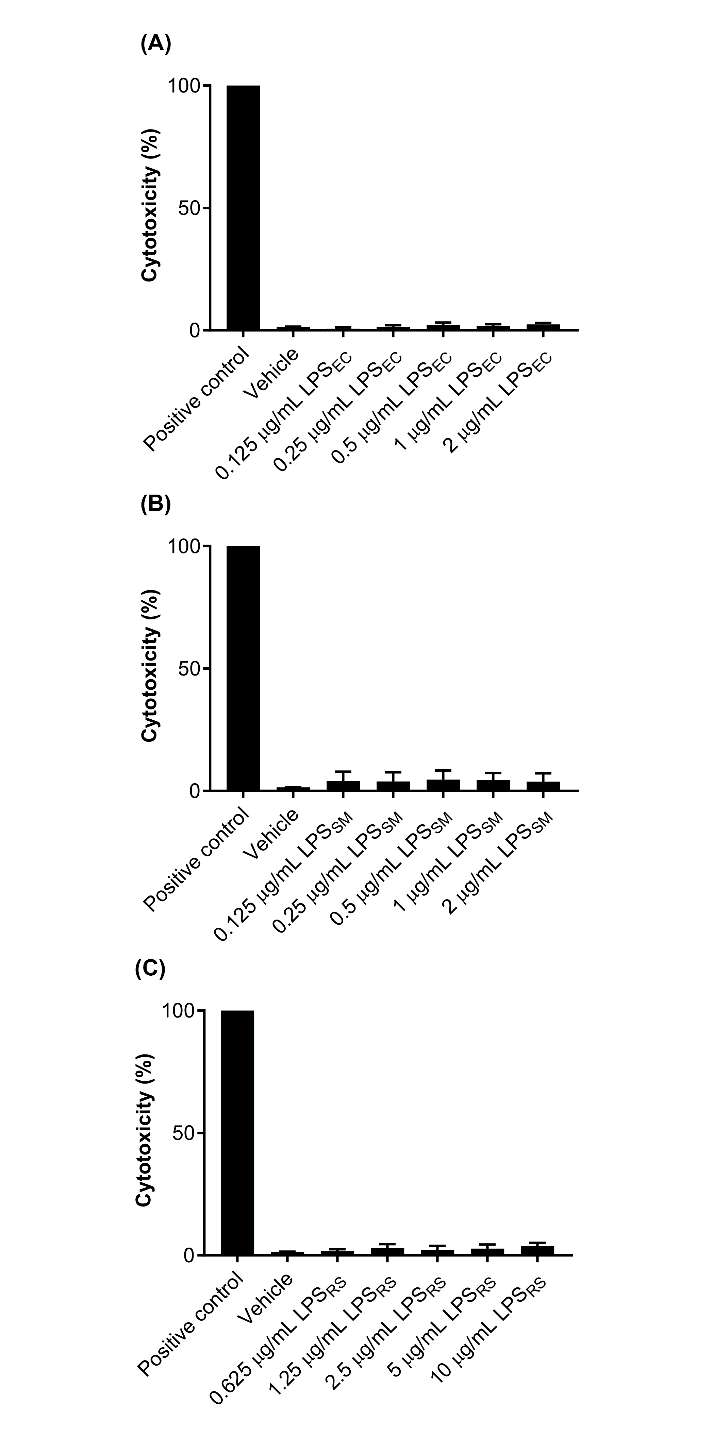


Figure S5: ***Cytotoxic effects of LPS chemotypes in platelets.*** The cytotoxic effects of LPS_EC_ (**A**), LPS­_SM_ (**B**), and LPS_RS_ (**C**) in platelets were investigated using a lactate dehydrogenase (LDH) cytotoxicity assay. All the results were normalised to the positive control. Data represent mean ± S.D. (n=3) and analysed with one-way ANOVA with Dunnett’s post-hoc test.


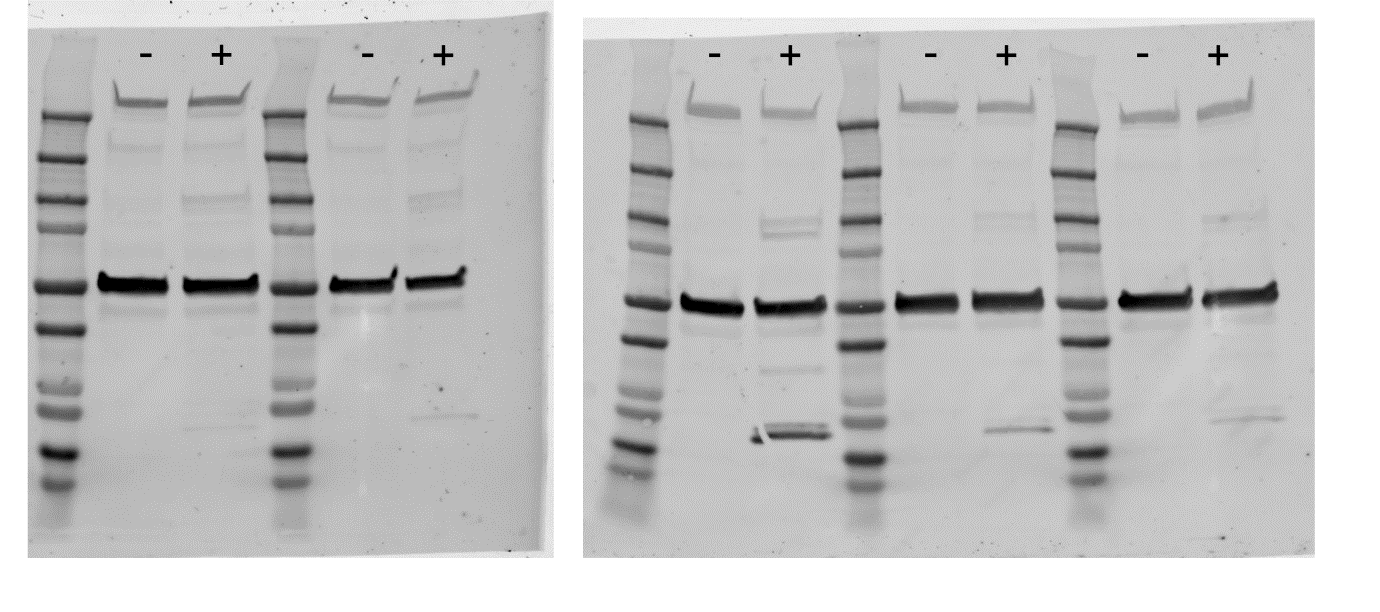


Figure S6: **Full length immunoblots of TLR4 and α-tubulin in whole platelet lysates shown in Figure 2.** Resting (-) and CRP-XL (0.5μg/mL) activated (+) platelets were examined for the presence of TLR4 via immunoblotting. The level of α-tubulin was detected as a loading control.
